# Regulation of PDE5 expression in human aorta and thoracic aortic aneurysms

**DOI:** 10.1038/s41598-019-48432-6

**Published:** 2019-08-21

**Authors:** Valeriana Cesarini, Calogera Pisano, Gabriele Rossi, Carmela Rita Balistreri, Flavia Botti, Giorgio Antonelli, Giovanni Ruvolo, Emmanuele A. Jannini, Susanna Dolci

**Affiliations:** 1 Department of Biomedicine and Prevention, University of Rome TorVergata, Rome, Italy; 2 Saint Camillus International University of Health and Medical Sciences, Rome, Italy; 3 Department of Surgery Sciences, University of Rome TorVergata, Rome, Italy; 4 Department of Biomedicine, Neuroscience and Advanced Diagnostics (Bi.N.D.), University of Palermo, Palermo, Italy; 5 Department of Clinical Sciences and Translational Medicine, University of Rome TorVergata, Rome, Italy; 6 Department of Systems Medicine, University of Rome TorVergata, Rome, Italy

**Keywords:** Risk factors, Angiogenesis

## Abstract

Aneurysms and dissections affecting thoracic aorta are associated with smooth muscle cell (SMC) dysfunction. NO/cGMP signaling pathway in smooth muscle cells has been shown to be affected in sporadic thoracic aortic aneurysms. We analyzed the mRNA levels of PDE5, a cGMP-hydrolyzing enzyme highly expressed in aortic SMCs, that regulates arterious vascular tone by lowering cGMP levels. We found that aortic tissue obtained from Marfan, tricuspid and bicuspid thoracic aneurysms expressed lower levels of PDE5 mRNA compared to control aortas. In particular, we found that affected aortas showed lower levels of all the PDE5A isoforms, compared to control aortas. Transfection of vascular SMCs (VSMCs) with NOTCH3 activated domain (NICD3) induced the expression of PDE5A1 and A3 protein isoforms, but not that of the corresponding mRNAs. VSMC stimulation with GSNO, a nitric oxide analogue or with 8-br-cGMP, but not with 8-br-cAMP, up-regulated PDE5 and NOTCH-3 protein levels, indicating a negative feedback loop to protect the arterial wall from excessive relaxation. Finally, we found that PDE5 is expressed early during human aorta development, suggesting that if loss of function mutations of PDE5 occur, they might potentially affect aortic wall development.

## Introduction

Thoracic aortic aneurysm (TAA) and dissection (TAD) remain a significant clinical challenge due to their high morbidity and mortality^1,2^. In the United States, diseases of the aorta account for more than 40.000 deaths per year^3^. The aetiology of TAA is heterogeneous and can be classified in syndromic and non-syndromic forms. The first includes inherited and familiar TAA that characterizes younger patients. The second group includes sporadic or isolated TAAs, that occur with aging. Inherited TAAs have an incidence of 5% and are represented by Marfan syndrome, Ehlers-Danlos syndrome, Loeys-Dietz and aneurysm-osteoarthrosis syndrome^4^. Within the TAAs, the familiar forms have a 20% incidence and are represented by TAA associated with bicuspid aortic valve (BAV), patent ductus arteriosus (PDA) and cerebro-vascular disease. Isolated or sporadic TAAs, accounting for the remaining 75%, have a degenerative etiology linked to classic cardiovascular risk factors, such as smoking, hypertension, and dyslipidemia^5,6^ and are defined as tricuspid aortic valve aneurysms (TAV). Recent studies showed a genetic basis of all forms of TAA even in the sporadic type^7^. It is predominantly inherited in an autosomal dominant fashion with reduced penetrance and variable expression, showing significant genetic (>20 gene) and clinical (location, severity and age onset) heterogeneity^8^. Most genes associated with the development of TAA encode proteins involved in the extracellular matrix maintenance, SMC contraction or metabolism, or in signaling pathways^9^. The causative genes have been grouped into several categories: structural and regulatory proteins involved in SMC contractile function^10–14^, proteins involved in the maintenance of or SMC adhesion to the extracellular matrix^12,15^, proteins involved in canonical TGF-β or NOTCH1 signaling^16–23^ and proteins involved in SMC metabolism or survival^14,24^. Sporadic TAAs have been linked to altered cGMP activated pathway and deregulated nitric oxide synthase (NOS) has been involved in TAA degeneration^25–27^. In addition, a single heterozygous mutation in the gene encoding cGMP-dependent protein kinase 1 (PRKG1; protein PKG-1; chromosomal 10q11.2–q21.1) has been demonstrated to cause sporadic TAA. PKG-1 is activated on binding of cGMP and plays an important role in SMC relaxation. The causal mutation, pArg177Gln, abolishes binding of cGMP and constitutively activates PKG1. Individuals harbouring this mutation have TAD at a relatively young age (15–51 years) at diameter ranging 4.3 to 5.7 cm^14,28^.

Under physiological conditions, cGMP signaling is strictly modulated by cGMP synthesis and inactivation. This latter is carried out by cGMP-phoshodiesterases (cGMP-PDEs) that can specifically hydrolize cGMP or both cGMP and cAMP^29^. The most abundant cGMP-PDEs present in arterial smooth muscle are PDE1A, 1B, and 1C, PDE3A and 3B, and PDE5^29^. Of these enzymes, PDE5 has attracted the attention of researchers during the recent years, because it represents the recognized target in treating erectile dysfunction (ED) and several other dysfunctions and symptoms. PDE5 is highly expressed in smooth muscle cells of vessels and as for other PDEs, three isoforms have been identified in humans including *PDE5A1*, *PDE5A2*, and *PDE5A3*^30–32^. These RNAs differ in the 5′ terminus of the respective mRNAs and the transcripts originate from three alternative first exons in the pre-mRNA. All the three isoforms, that show differential amino acid lengths (PDE5A1 is 875, PDE5A2 is 833, and PDE5A3 is 823 amino acid long) have the same cGMP catalytic activity and are sensitive to PDE5 specific inhibitors^33^.

NOTCH superfamily contains four members, NOTCH1, NOTCH2, NOTCH3 and NOTCH4, that encode for transmembrane proteins that activate an intracellular signaling pathway through interactions with adjacent ligand expressing cells that lead to the regulation of cell survival, proliferation and differentiation^34^. NOTCH signaling plays an essential role in the regulation of smooth muscle differentiation, and mutations or haploinsufficiency of NOTCH1 or NOTCH3 have been shown to be involved in a subset of TAA^35,36^ or in cerebral aneurysms, respectively. NOTCH1 signaling has been shown to be positively influenced by cGMP levels and PDE5 inhibition by sildenafil increases stemness of glioma cancer initiating cells *in vitro* and *in vivo*^37^. Considering the role of PDE5 in regulating the level of cGMP in smooth muscle cells, in this pilot study we sought to analyse PDE5, NOTCH1 and NOTCH3 expression in TAA patients with Marfan’s Syndrome, BAV and TAV. We also studied if NOTCH overexpression can affect PDE5 expression *in vitro* in smooth muscle cells and in glioma cells. Finally, to understand if PDE5 can play a role in aorta development, we studied its ontogenetic expression profile during early fetal development, both in mice and humans.

## Results

### PDE5 is the most abundant cGMP-PDE in adult human aortas and its expression is strongly down-regulated in TAAs

**Figure 1:**
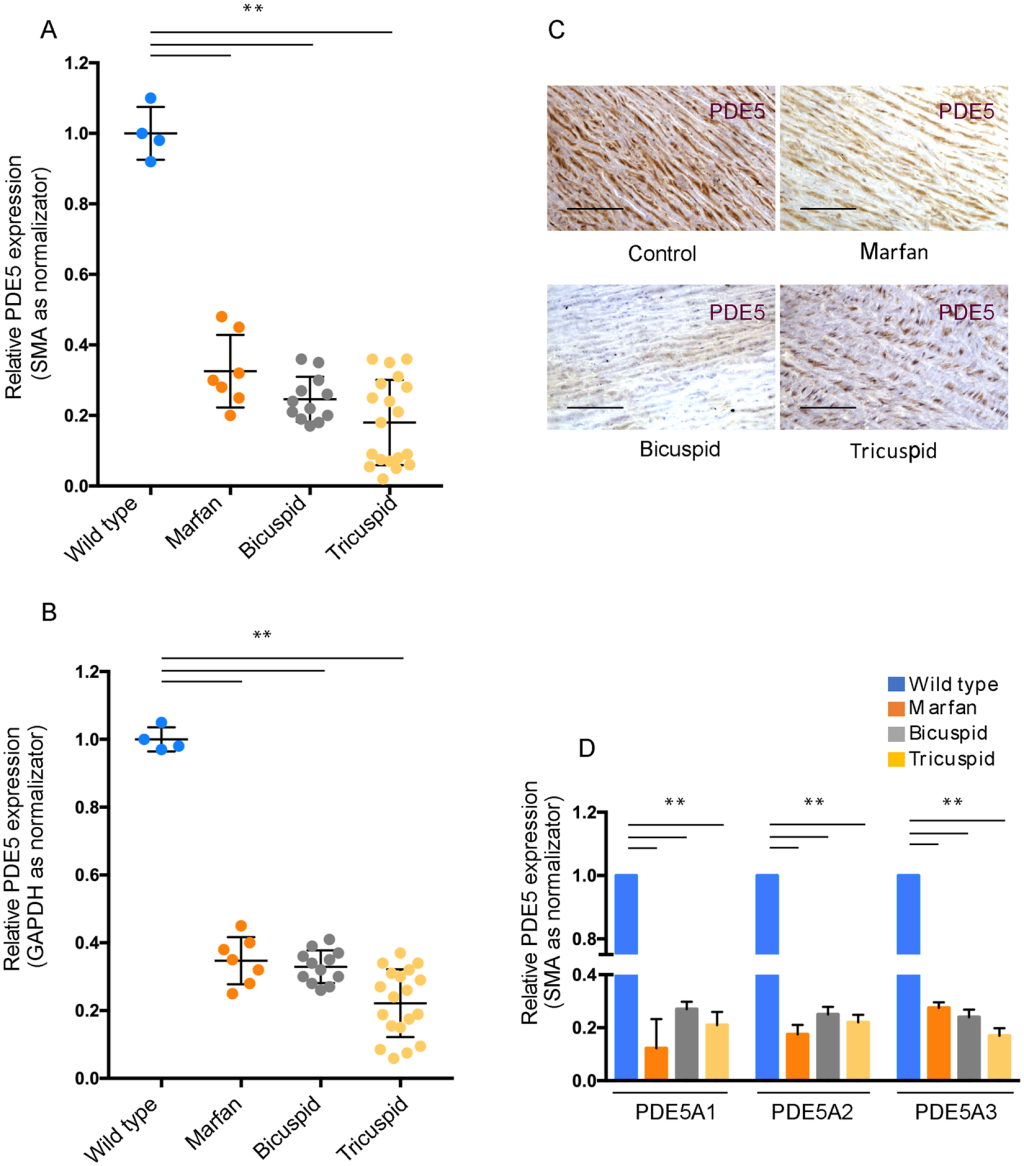
PDE5 expression in control aortas and in TAAs samples. Quantitative RT-PCR showing *PDE5* mRNA levels downregulation in TAV, BAV and Marfan syndrome TAA samples compared to control aortas. Normalization of *PDE5* mRNA levels was referred to *SMA* (**A**) and *GAPDH* (**B**) mRNA levels. Data were normalized to the mean of control values, set to 1.0. (**C**) Representative images of PDE5 expression in the aortic medial layer of control, Marfan, BAV and TAV samples by immunoistochemistry. Bars = 50 μm (**D**) Quantitative RT-PCR of *PDE5A1*, *A2* and *A3* isoforms in controls and TAA specimens. Bars represent ± SD. **p < 0.01 on three experiments.

Since cGMP metabolism defects are associated with familial TAA syndromes and PDE5 is highly expressed in SMCs of the aortic wall, we hypothesized that a correlation between PDE5 expression and medial defects might exist. By qRT-PCR we evaluated total *PDE5* mRNA levels in TAV, BAV and Marfan syndrome TAA samples and compared them to control aortas. Normalization of *PDE5* mRNA was referred either to *smooth muscle actin* (*SMA)* mRNA levels, since PDE5 is expressed exclusively in smooth muscle cells and their number could be altered in TAAs or to *GAPDH*. As shown in Fig. 1 we found that *PDE5* mRNA levels were strongly decreased in all the three pathological conditions compared to control aortas (Fig. 1A,B) either when normalizing for *SMA* or for *GAPDH* levels. Moreover, any statistically significant difference of the decrease of *PDE5* levels among the three types of TAA samples could be observed (Fig. 1A,B). Accordingly, by immunohistochemistry we found that PDE5 staining in the medial layer of the aortic samples was significantly decreased in all the three pathologies compared to control samples (Fig. 1C). As a negative control for PDE5 immunohistochemistry we omitted the primary antibody in the staining procedure (Suppl. Fig. 1E). PDE5A expression is characterized by the presence of three isoforms, A1, A2 and A3, whose expression has not very well characterized in human tissues. While *PDE5A1* and *A2* first exons are alternative exons, *PDE5 A3* first exon spans the first intron and second exon of *PDE5 A1* isoform. To correctly analyze their expression in control and aneurysm aortas, we performed qRT-PCR analysis using isoform specific forward primers (exon 1 sequence for the *PDE5A1* isoform, alternative exon 1 sequence for the *PDE5A2* isoform and intron1/exon2 boundary sequence of for the PDE5A3 isoform) and a common reverse primer within exon 3. We found that all the three *PDE5* isoforms (*PDE5A1*, *A2*, *A3*) were decreased in the pathologic samples compared to control aortas (Fig. 1D).

We also evaluated the expression levels of other cGMP-hydrolizing phosphodiesterases such as *PDE1A*, *PDE2A PDE3A* and *PDE9* in control and TA aortas, however their levels were at the limit of the detection threshold in all the tissue analyzed and thus not assayable (not shown).

### NOTCH1 and NOTCH3 levels expression in TAAs

**Figure 2:**
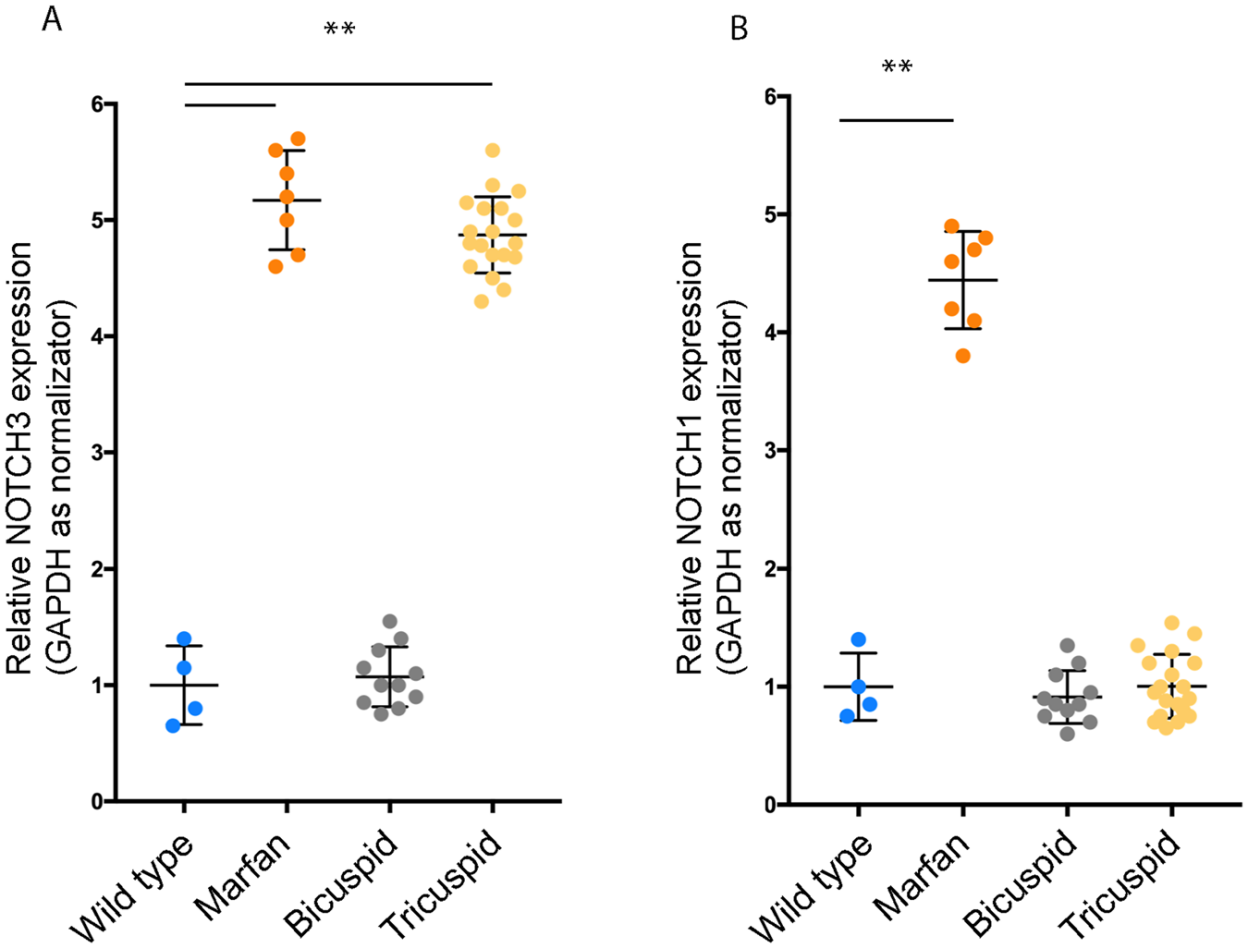
NOTCH1 and NOTCH3 expression levels in TAAs. Quantitative RT-PCR analysis showing *NOTCH3* mRNA levels increase in Marfan and in TAV samples compared to BAV samples and control aortas (**A**) and *NOTCH1* mRNA levels increase in Marfan syndrome samples compared to BAV, TAV and control samples (**B**). Data were normalized to the mean of control values, set to 1.0. Bars represent ±SD. *p < 0.01 on three experiments.

The regulation of PDE5 expression is still an open question and no results have been reported to date. Thus, we investigated if PDE5 expression might be correlated to the expression of transcription factors that are involved in aortic aneurysm and/or smooth muscle development. To this end, we identified two components of the NOTCH family, NOTCH1 and NOTCH3, whose mutations are involved in abnormal VSMCs development^38–40^. By quantitative RT-PCR analysis we found that *NOTCH3* levels were increased in Marfan and in TAV samples but not in BAV samples (Fig. 2A). Also *NOTCH1* levels were increased in Marfan syndrome samples, however they were similar to control samples in BAV and TAV samples (Fig. 2B). We noticed that one of out 12 BAV samples was completely negative for *NOTCH1* expression, but not for *NOTCH3*, and separated it from the remaining BAV samples (not shown). Since the major source of *NOTCH1* expression comes from the endothelial compartment of the arterial wall, it is possible that a reduced contribution of endothelium in this sample was the cause of reduced *NOTCH1*, but not *NOTCH3* levels.

### PDE5 expression is up-regulated by NOTCH3 and down-regulated by NOTCH1

**Figure 3:**
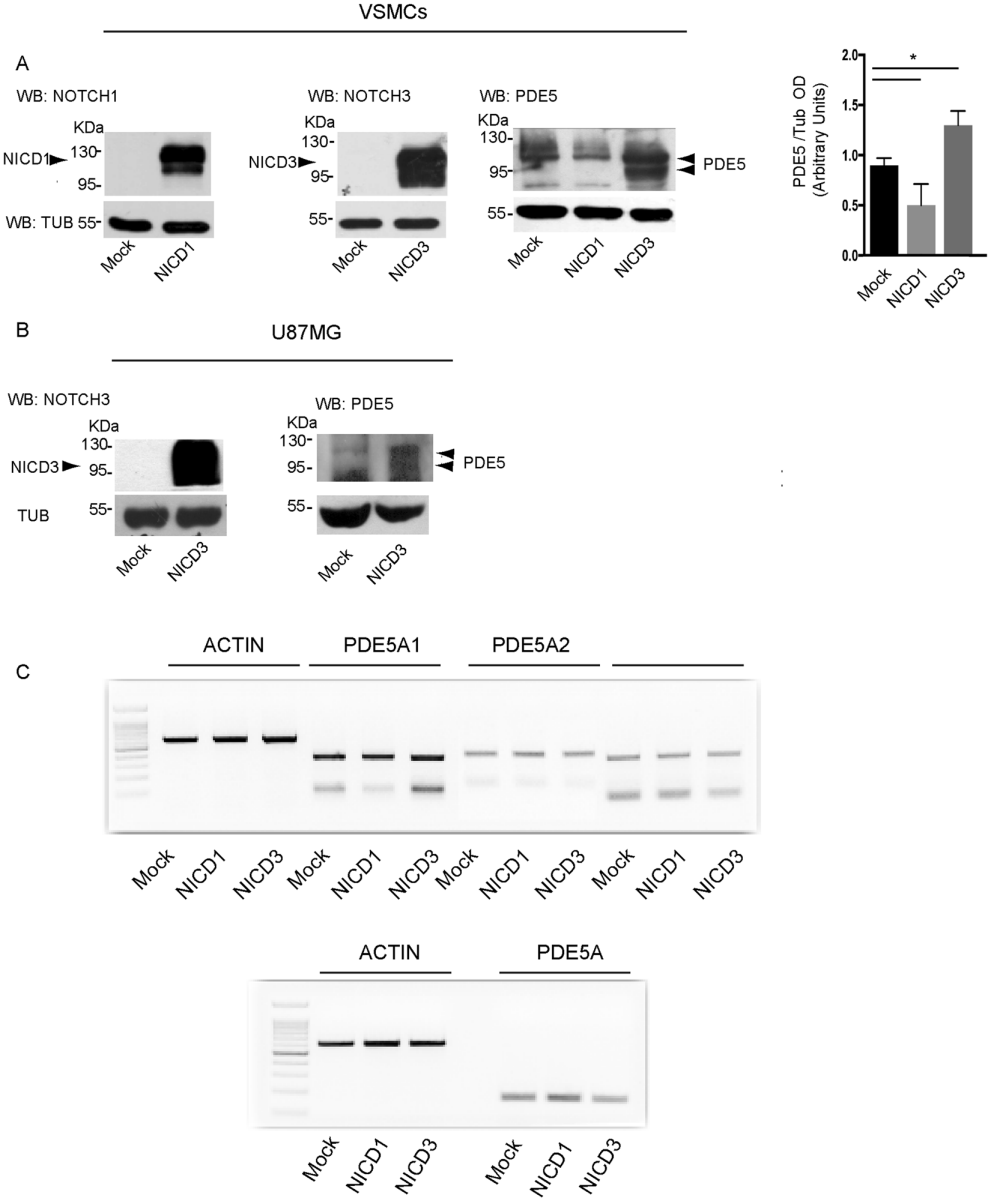
NOTCH3 regulates PDE5 expression. Representative western blot showing decrease of PDE5 protein levels in human VSMCs transfected with NICD1 and increase of PDE5 expression upon NICD3 intracellular domain transfection in human VSMCs (**A**) and in U87MG cells (**B**). Bottom panel in (A) represents densitometric analysis on four independent experiments. Bars represent ±SD *p < 0.05. Representative semi-quantitative RT-PCR showing mRNA levels of total *PDE5A, PDE5A1, A2* and *A3* isoforms in NICD1 or NICD3 overexpressing human VSMCs (**C**). Three independent experiments were performed.

Since we found that NOTCH1 and NOTCH3 levels are differentially expressed in the aortic aneurysm samples, we next evaluated if they might regulate PDE5 expression in VSMCs *in vitro*. To this end we performed overexpression experiments by transfecting the intracellular domain of NOTCH1 (NICD1) or NOTCH3 (NICD3) in uterine VSMC. We found that NICD1 transfection induced a significant decrease of PDE5 protein levels in VSMCs (Fig. 3A) while NICD3 overexpression, on the contrary, induced a significant up-regulation of the intensity of PDE5 A1 band (Fig. 3A) and the appearance of an extra band of approximately 85 KDa of molecular weight (MW), compatible with the MW of the PDE5A3 isoform. Similarly to VSMCs, also U87MG cells, a glioblastoma cell line that expresses low levels of PDE5, up-regulated both the A1 and A3 isoforms when transfected with NOTCH3 (Fig. 3B), suggesting a common mechanism of NICD3 action on PDE5 expression in smooth muscle and neural cell types. Thus, to understand if NOTCH1 and/or NOTCH3 were acting at transcriptional level, we evaluated *PDE5* mRNA levels in NICD1 or NICD3 overexpressing VSMCs. As shown in Fig. 3C we found that the mRNA levels for *PDE5A1*, *A2*, *A3* isoforms and for total *PDE5* did not significantly change following NICD1 or NICD3 transfection, indicating that their opposing effects on PDE5 protein levels were not occurring at the transcriptional level in VSMCs.

### An increase of intracellular cGMP but not cAMP induces PDE5 up-regulation

**Figure 4:**
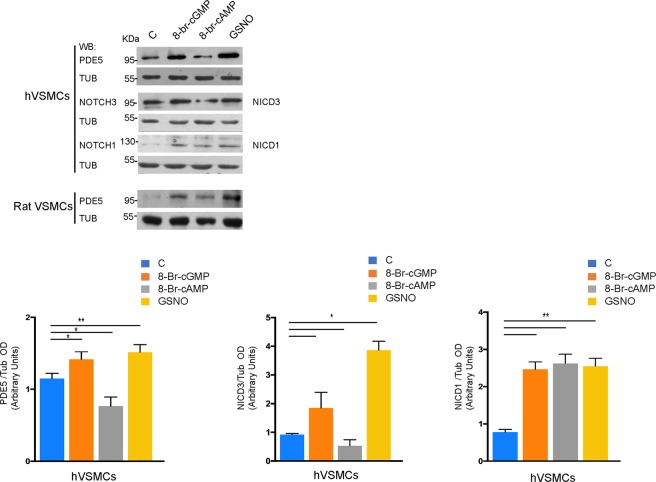
PDE5 expression after cGMP or cAMP stimulation of human and rat VSMCs. Representative western blot showing PDE5, NOTCH1 and NOTCH3 protein levels in human VSMCs (**A**) and PDE5 protein levels in rat VSMCs 24 hr post treatment with 0.5 mM 8-Br-cGMP, 0.5 mM 8-Br-cAMP and 100 nM GSNO respectively (**B**). Histograms represent densitometric quantification of western blot analysis in (**A**). Bars represent ±SD. *p < 0.05 on three experiments.

Both on demand and chronic use of PDE5 inhibitors to treat erectile dysfunction or pulmonary hypertension, that lead to the increase of intracellular cGMP levels, has raised potential safety issues for the rupture of pre-existing aneurysms^41^. Since PDE5 expression is low in aortic aneurysms, we tested if high levels of intracellular cGMP were able to modulate PDE5 expression. We treated VSMCs from human or rat (RCC) origin for 24 hr with 0.5 mM 8-Br-cGMP or with 0.5 mM 8-Br-cAMP (membrane-permeable analogs of the second messengers cGMP and cAMP, respectively) and in parallel experiments we stimulated VSMCs with 100 nM GSNO, a NO donor that stimulates the soluble guanylate cyclase to produce cGMP. As shown in Fig. 4, we found that 8-br-cGMP or GSNO sensibly up-regulated PDE5 expression while 8-Br-cAMP, that is physiologically generated by β_2_-adrenergic receptor stimulation of the medial layer, down-regulated PDE5 levels both in human and rat smooth muscle cells. We also found that NICD1 and NICD3 were up-regulated in human VSMCs. However NICD3, similarly to PDE5, was significantly down-regulated by high intracellular cAMP levels.

### Developmental expression of PDE5 in mouse and human fetal aortas

**Figure 5:**
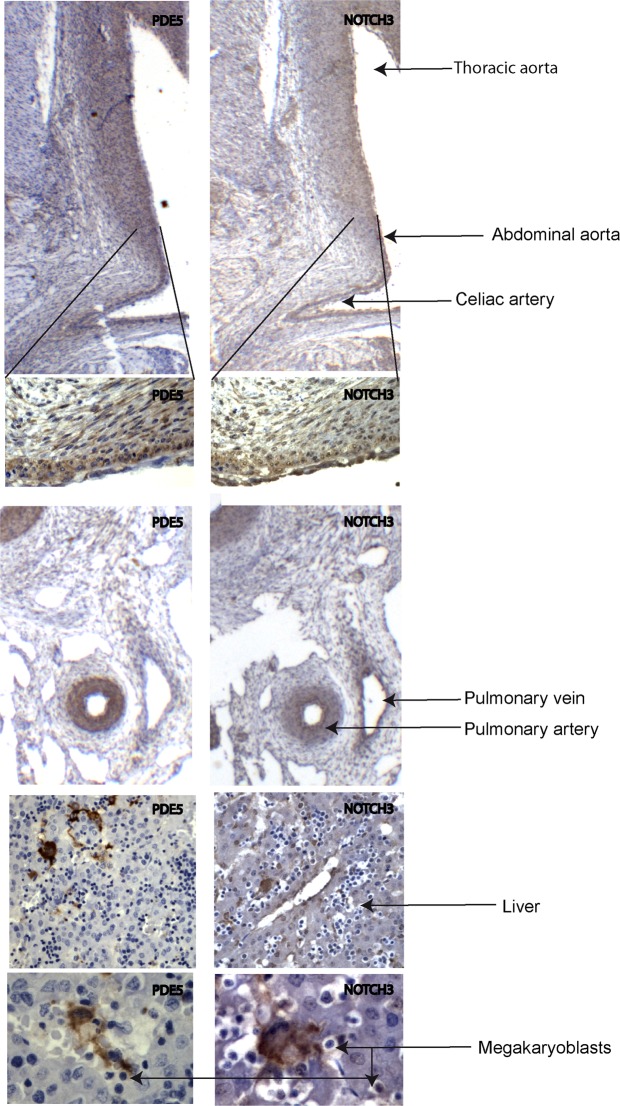
PDE5 and NOTCH3 expression during development of mouse and human fetal aortas. Representative immunohistochemical evaluation of PDE5 and NOTCH3 on early mouse (12 days post coitum, dpc) and human (7 weeks post-conception, wpc) embryos showing positivity for both protein in the medial layer of the dorsal aorta, pulmonary arteries, gastrointestinal tract, and in megakaryoblasts of the liver in both mouse and human embryos. Black arrows indicate thoracic aorta, abdominal aorta, celiac artery, pulmonary vein and pulmonary artery respectively.

Despite the plethora of data on PDE5 expression and activity in normal and pathological human adult tissues, data are missing on its expression in human or even in animal model embryos. To study the tissue distribution and the developmental stage of PDE5 expression and to co-localize it with NOTCH3 during early development, we performed PDE5 and NOTCH3 IHC experiments on early mouse and human embryos. We found that PDE5 was barely expressed in early embryos from both species (12 days post coitum, dpc, in the mouse and 7 weeks post-conception, wpc, in the human, 2 embryos/species observed), however discrete areas were positive to anti-PDE5 antibodies either in mouse or in human embryos. In the mouse embryo PDE5 was mainly localized in the mesenchymal tissue included between the dorsal aorta and the anterior gut and in sparse cells within the liver bud (Suppl. Fig. 1A). Similarly to the mouse, positive PDE5 staining was found in the mesenchyme localized between the dorsal aorta and the primitive intestine in the human embryo (Suppl. Fig. 1B). At a later human embryonic stage (12 wpc, 2embryos observed) PDE5 expression was still confined to few tissue types. Strong PDE5 positivity was found in the medial layer of the thoracic and abdominal aorta (included collaterals), pulmonary arteries and gastrointestinal tract, and in sparse polynucleated cells of the liver (Fig. 5A). These cells proved to be megakaryoblasts, as identified by anti-Von Willebrand antibody in *in vitro* cultures of mouse bone marrow cells (Suppl. Fig. 1D), in agreement with the notion that human platelet are one of the cellular sites of the highest PDE5 activity^42^. Analysis of NOTCH3 revealed that its expression was overlapping that of PDE5 in the inner smooth muscular layer of the dorsal aorta (Fig. 5B) and primitive intestine (Suppl. Fig. 1D) and interestingly, also in megakaryoblasts of the liver (Fig. 5C).

## Discussion

Vascular smooth muscle cells, that physiologically control the vascular tone of the arterial bed, counteract hypertensive stimuli to undergo relaxation by increasing intracellular cyclic GMP and AMP nucleotides. Endogenous vasodilators such as nitric oxide (NO), atrial, brain or type C natriuretic peptides (ANP, BNP and CNP, respectively) control smooth muscle tone through activation of guanylyl cyclase, elevation of cGMP, and activation of cGMP-dependent protein kinase (PKG). PKG then phosphorylates physiological substrates to reduce intracellular Ca^2+^ concentration and to desensitize cells to Ca^2+^, leading to smooth muscle relaxation^29^. PDE5 is the main cGMP-hydrolizing PDE (cGMP-PDE) that lowers cGMP concentration in aortic smooth muscle cells and together with PDE1A, a Calcium-Calmodulin depentent cGMP-PDE, revert the relaxation state^29^. Genetic alterations of factors that regulate the NO/cGMP pathway have been shown to occur in a subset of familiar thoracic aortic aneurysms, indicating that unbalanced cGMP-dependent cascade plays an important role in the generation of aortic wall defects. In support to this evidence is the observation that NO synthase2 (NOS2), one of the five NO synthetic enzymes, is a key mediator of the aortic pathology found in Adamts1 haploinsufficiency and in Marfan syndrome^26^. In these contexts PDE5 may prevent excessive smooth muscle relaxation and aortic dilation resulting from prolonged stimuli that increase cGMP levels and cGMP-activated signaling pathway.

We previously showed that PDE5 expression *in vitro* is strongly increased and associated to quiescent, contractile SMCs and its levels are down regulated when VSMCs resume proliferation^43^, suggesting that PDE5 is a marker of the contractile phenotype. In agreement with these evidences, reports have demonstrated that switching of SMCs from the physiological contractile phenotype to a synthetic state associates to aortic aneurysm formation and rupture^44^, suggesting that PDE5 lowering in contractile SMCs might play an important role in the genesis of aortic aneurysms. In line with this hypothesis, here we report that PDE5 protein and mRNA levels are significantly decreased in the wall of Marfan, BAV and TAV aortic aneurysms compared to control aortas, thus associating PDE5 to aortic aneurysm development. The decrease of *PDE5* total mRNA levels in aortic aneurysm tissue corresponded to the mRNAs decrease of all the three isoform that, on the contrary, were normally expressed in control aortas.

Our results suggest that PDE5 might play an important role in the regulation of cGMP levels in VSMCs. In mouse cardiomyocytes it has been recently shown that the three PDE5A isoforms had differential subcellular localization, PDE5A1 being mainly localized in the cytoplasm, while mPDE5A2 and mPDE5A3 showing also nuclear distribution^30^. We previously demonstrated that PDE5 is mainly localized in the cytoplasmic compartment and also concentrated within the centrosomes of myometrial SMCs^43^, strongly supporting its role in contraction, and possibly in the control of cell division. The lack of isoform specific antibodies, however, makes it difficult to identify the correct localization of these isoforms in human cells. We found that PDE5 is expressed in the aortic smooth muscle layer of early mouse and human embryos, suggesting that cGMP metabolism during aortic development is active and might play a role in the control of the arterial wall development and function.

Although it has been hypothesized that *PDE5* transcripts arise from two different promoters and/or by the splicing of three alternative first exons in the pre-mRNA^33^, very few is known on the regulation of their expression in human tissues. Among the potential factors that could regulate the synthesis of *PDE5* mRNA isoforms in smooth muscle cells, we found that components of the NOTCH family of transmembrane/co-transcription factors involved in VSMCs development were differentially expressed in aortic aneurysm samples and that they could influence PDE5 expression in smooth muscle cells. We found that *NOTCH3* expression was increased in Marfan syndrome and TAV samples while *NOTCH1* was up-regulated only in Marfan syndrome aortas, suggesting that the defects of extracellular matrix structure present in this syndrome can affect NOTCH expression. By overexpressing constitutive active intracellular domains of NOTCH1 or NOTCH3 we observed that they differently modulated PDE5 levels in VSMCs *in vitro*. While NOTCH1 overexpression induced a decrease of PDE5 levels, NOTCH3 up-regulated PDE5A1 and PDE5A3. The modulation of PDE5 protein expression was not mediated by modification of mRNA levels for none of the isoforms, as indicated by semiquantitative RT-PCR experiments. While we do not think that NOTCH1 can control PDE5 expression cell-autonomously *in vivo*, since it is primarily expressed in the endothelial compartment, our results suggest that NOTCH3 might affect PDE5 expression by controlling mRNA translation of the specific PDE5 isoforms or by affecting protein stability and its degradation. In support to this latter hypothesis, a recent report has identified a PDE5 ubiquination system, based on RhoBTB1, a Cullin3-binding protein that controls PDE5 ubiquitination, its stability and its enzymatic activity^45^. The evidence that *NOTCH3* mRNA levels are not modified in BAV compared to control tissues while in Marfan syndrome and TAV aortic specimens they are even increased, does not rule out the possibility that NOTCH3 activation by proteolytic cleavage that leads to NOTCH-activated signaling pathway is impaired and/or not working in the different aneurysmatic pathologies. Moreover, the evidence that PDE5 expression parallels that of NOTCH3 mapping to the same smooth muscle structures, as well as in megakaryocytes, during human embryonic development suggests a link between these two proteins.

Specific PDE5 inhibitors are currently under clinical trial during prenatal life for the treatment of fetal growth restriction (FGR), neonatal pulmonary hypertension or preeclampsia^46–48^ to target vascular smooth muscle layers, thus the safety of these molecules on the development of the aortic wall needs to be warranted. We found that *in vitro* stimulation of VSMCs with a membrane-permeable cGMP analog up-regulates PDE5 levels, indicating that a negative feedback control tightly monitors cGMP levels in smooth muscle cells. On the contrary, increased intracellular cAMP concentrations decrease PDE5 levels, potentially amplifying the mio-relaxant effects of cAMP. The same effects of cGMP and cAMP were observed to regulate activated NICD3 levels, suggesting that malfunction of cGMP/NICD3/PDE5 loop and excessive VSMC relaxation could contribute to aortic aneurysm formation. In conclusion, we suggest that PDE5 expression and activity might play a protective role in the NO/cGMP/PKG axis by controlling the vascular tone of SMCs. It will be interesting to verify this hypothesis on *Pde5* KO animal models.

## Materials and Methods

### Populations

This study protocol was approved by the institutional review board at University of Rome TorVergata. It included 38 TAA individuals (7 patients with Marfan’s Syndrome, 19 TAV patients and 12 BAV patients). They were admitted in the Cardiac Surgery Unit of Tor Vergata University from July 2017 to December 2018 for ascending aorta surgical procedure. Control aortas without aortic pathologies were obtained from organ donors from the organ transplantation center (University of Torvergata). To match the age of control donors we chose 2 young donors (37 and 45 years of age, male and female, respectively) and from 2 older donors (68 and 78 years of age male and female, respectively) to match them with the patient age. Our study received approval from local ethic committees and all participants gave their informed consent. Data were encoded to ensure patient and control protection. All demographic variables, echocardiographic parameter and operative characteristics are summarized in Table 1.

**Table 1:** Demographic characteristics, echocardiographic parameter, operative characteristics of the study population.

Variables	Marfan Syndrome (n = 7)	Tricuspid Valve (n = 19)	Bicuspid Valve (n = 12)	p-value
BSA	1.9 [1.2, 2.4]	1.9 [1.5, 3.3]	1.9 [1.5, 2.1]	0,875
BMI	21.9 (5)*	28.5 (4.3)*	25.8 (2.5)	0,002
Age	35.9 (14.5)*	69.4 (10.5)*	61 (12.9)*	<0.001
Gender M	4 (57.1%)	13 (68.4%)	10 (83.3%)	0,357
Hypertension	5 (71.4%)	13 (68.4%)	10 (83.3%)	0,628
Familiarity for cardiovascular diseases	7 (100%)	14 (73.6%)	9 (75%)	0,438
**Echocardiographyc parameter**				
Aortic root	50 [44, 55]	47 [32, 53]	44.5 [35, 50]	0,078
Ascending aorta	47.4 (6.8)	50.4 (3.7)	47.3 (4.8)	0,152
Left ventricul telediastolic diameter	51.4 (3.2)	51.1 (4.8)	50.8 (3.2)	0,938
Left ventricul telesistolic diameter	36 [33, 40]	36 [24, 43]	36 [28, 38]	0,867
Septum	11 [10, 12]	11 [9, 14]	12 [10, 16]	0,221
Posterior wall	11 (0.8)	11.1 (1.7)	12 (1.9)	0,259
Left ventricul telediastolic volume	96.9 (29.7)	110.1 (20.3)	100.9 (22.2)	0,342
Left ventricul telesystolic volume	53.1 (9.1)	53.8 (9.4)	47.7 (9.8)	0,206
Ejection Fraction%	55 [55, 70]	55 [50, 60]	58 [50, 60]	0,885
Mitral regurgitation (I or II)	5 (71.4%)	15 (78.9%)	6 (50%)	0,269
Aortic stenosis	0 (0%)	2 (10.5%)	6 (50%)	0,017
Aortic regurgitation	3 (42.8%)	9 (47.3%)	5 (41.6%)	1,000
Systolic pulmonary arterial pressure	29.1 (6.2)	31.2 (6.8)	31.1 (5.6)	0,747
**Operative characteristics**				
Procedure type				0,280
*Bental-De Bono*	7 (100%)	14 (73.6%)	11 (91.6%)	
*Ascending aorta replacement*	0 (0%)	5 (26.3%)	1 (8.3%)	
Associated procedures, yes	2 (28.5%)	5 (26.3%)	1 (8.3%)	0,498
Emergency	0 (0%)	1 (5.2%)	2 (16.6%)	0,568
Type of aortic prostheses				0,081
*mechanical*	7 (100%)	10 (52.6%)	7 (58.3%)	
*bioprosthesis*	0 (0%)	9 (47.4%)	5 (41.7%)	
Size of aortic prostheses	23 [19, 25]	23 [19, 27]	23 [21, 25]	0,878
Size of vascular prostheses	25.9 (1.5)	27.3 (1.9)	26.7 (1.3)	0,166
Cardioplegia				0,805
*Cristalloid*	7 (100%)	16 (16%)	11 (91.7%)	
*Ematic*	0	3 (15.7%)	1 (8.3%)	
Surgery time	310 [210, 800]	240 [160, 540]	232 [185, 393]	0,093
Ejection Fraction %	60 [35, 70]	57 [40, 60]	60 [42, 65]	0,537

### Embryos, Aortic specimens and histopathological assays

Three human fetuses were obtained from pregnant women who underwent therapeutic abortion at 9–12 weeks of gestation at the Dipartimento di Promozione della Salute, Materno-Infantile, Medicina Interna e Specialistica di Eccellenza “G. D’Alessandro” (PROMISE) of the University of Palermo. The ethical committee of the Department of Biomedicine, Neuroscience and Advanced Diagnostics of the University of Palermo, Italy approved the use of human fetal tissue for research purpose. The study was performed in accordance with the relevant guidelines and regulations. Informed, written consent was obtained from all patients. Mouse embryos were obtained from CD-1 pregnant females at 12 and 15 days post coitum (dpc). All experiments were performed in compliance with the Tor Vergata University Institutional Animal Care and all the procedures adhered to the standards published in Guide for the Care and Use of Laboratory Animals. Experimental procedures involving mice were approved by the Italian Ministry of Health. For immunolocalization studies, human and mouse fetal tissues were fixed in formalin and embedded in paraffin. Immunohistochemistry was performed on 5 μm sections. For PDE5A detection, de-paraffinized and rehydrated sections were retrieved in TE solution (pH 9). Endogenous peroxidase was blocked (ScyTek, Logan, UT) and then sections were incubated for 1 h at room temperature with rabbit polyclonal antibody anti-PDE5A (1:100 diluition, H-120, sc 32884; Santa Cruz Biotech, Heidelberg, Germany). This antibody has been raised against aminoacids 31–150 in the N-terminus of human PDE5, a region shared by PDE5A1, A2 and A3, and it is potentially able to recognize all the three PDE5 isoforms. NOTCH3 and VWF antigens were retrieved by microwave (10 min in EDTA buffer) and detection was performed with rabbit anti-NOTCH3 (1:100, cod. 5276, Cell Signaling, Milan, Italy) anti-VWF(A0082, Dako-Agilent, Milan, Italy). Retrieval HRP AntiPolyvalent Lab Pack (Scytek Laboratories, Utah, USA), (Alexa Fluor® 594, Thermofisher, Milan, Italy, 1:4000). DAB (Scytek Laboratories, Utah, USA) was used as chromogen in immunohistochemistry experiments. Sections were counterstained with Harris’s hematoxylin or DAPI. Morphology of control and TAA samples was performed by hematoxylin-eosin (H&E). For each control and patient aortic section, at least three immunostaining reactions were performed and four regions of interest (ROI) analysed. Control negative immunostainings were performed by omitting the primary antibody (see Suppl. Fig. 1E). Densitometric analyses were performed by ImageJ software analysis.

For RNA extraction, aortas were cut in squares of about 1 cm × 1 cm and snap frozen. Tissues or cultured cells were extracted with Trizol l reagent (Invitrogen). RNA was DNAase treated (RQ1 DNAase, Promega) and reverse transcribed with random hexamer primers using an Invitrogen cDNA reverse transcription kit (with a reverse transcriptase–negative control) cells by Quantitative real-time PCR was performed using SsoAdvanced™ Universal SYBR® Green Supermix (Biorad) on an Applied Biosystems 7300 Real Time Machine (Applied Biosystems). Relative expression was calculated using the 2^−DDCt^ method. CT means from samples for each condition were pooled and standard deviation (SD) was calculated.

Western blotting was performed on whole cell extracts obtained following cell lysis in 10 mM HEPES pH 7.9, 1% Triton X-100, 10 mM KCl, 1.5 mM MgCl2, 0.1 mM EGTA, 0.5 mM dithiothreitol (DTT), 10 mM β-glycerophosphate, 0.1 mM sodium vanadate, and protease inhibitor cocktail (Sigma-Aldrich, Milan, Italy). Rabbit anti-NOTCH1 (ab65297, Abcam, Cambridge, UK), rabbit  anti-NOTCH3 and mouse anti-Tubulin (T4026, Sigma-Aldrich) antibodies were diluted 1:1000. Signals were detected with HRP-conjugated secondary antibodies and enhanced chemiluminescence (Santacruz Biotech). Western blots were performed on three independent experiments.

### Cell culture and transfections

Uterine artery VSMCs were derived from young aged (25–35 years old women) myometrial samples obtained as reported in^43^ and consisted in explants of small fragments (0.5 mm squares) cut around the arteriolar regions. VSMC from uterine artery were used instead of aortic VSMCs because they showed a higher replating efficiency (more than ten passages) and a higher transfection efficiency (our unpublished results and^49^) but a lower infection efficiency with respect to tumor cell lines. The glioblastoma cell lines U87MG and T98G and Hek293T cells were cultured in DMEM 10%FCS. We included U87MG cells, that do not express PDE5, to show that PDE5 upregulation by NICD3 overexpression was not limited to VSMCs. To validate anti-PDE5 antibodies, T98G cells, that express high levels of PDE5, were infected with silencing vectors (sh-GFP as mock and sh-PDE5 as PDE5 silencing) as previously reported in^50^. Briefly, lentiviral PDE5 silencing vectors [RHS4430-101103886 Clone V3LHS_325328 (silencing sequence: CGGTTAATGCAGAAGTTG) sh-PDE5] and lentiviral scrambled control [RHS2349, sh-GFP] (Dharmacon, Milan, Italy) were transfected in Hek293T cells and all the lentiviral particles obtained were used undiluted for T98G infection.

Rat corpora cavernosa (RCC) smooth muscle cells (gift from E. Carosa) were obtained and cultured as reported in^51^. To prove that megakaryoblasts are the cell types in the fetal liver that express PDE5, we isolated them from mouse femoral bone marrow and cultured in Megacult medium (cat. 04961, Stem Cell Technologies, Cambridge, UK). 8-Br-cGMP, 8-Br-cAMP and GSNO were from Sigma-Aldrich (B1381, B7880 and N4148, respectively).

NICD1-pcDNA3 or NICD3-pcDNA3 plasmids (kind gift of prof. I. Screpanti) encoding for the intracellular domain (ICD) of each NOTCH protein or pcDNA3-empty plasmid (Mock), were transfected in uterine artery smooth muscle cells, using lipofectamine 2000 transfection reagent (Invitrogen, Milan, Italy).

### Oligonucleotides

**hPDE5 A1** For 5′-CGATCACTGGGACTTTACCT; **hPDE5 A2** For 5′-TGC TATGTTGCCCTTT GGAG; hPDE5 A3 For 5′-AACATGACGGAACCTTGCCA; **qPCR-hPDE5 A1-2-3** Rev 5′- TTATCTGCACGAGGACTCTG; **hPDE5 A1-2-3** Rev 5′-GAGCACTG GTCCCCTTCAT**; hSMA** For 5- ATGTACCCAGGCATTGCTG A; **hSMA** Rev 5′ TTGCTGAT CCACATCTGCTG; **hGAPDH** For 5′AGGTCGGAGTCAAC GGATTT; **hGAPDH** Rev 5′-GTGATGGCATGGACTGTGGT. **hNOTCH3** For 5′-GAATT GTGAAGTGAACGTGGA; **hNOTCH3** Rev 5′-TGGCACA GTCATCGATATTCT; **hNOTCH1** For 5′-TATTGACGACGTTGCCGGGTA; **hNOTCH1** Rev 5′ ATAGTCCTCGGATTG CCTG CACT.

### Statistical analysis

All analyses were performed with STATA 14.1 software. All categorical variables are reported as frequencies and percentages, while quantitative variables have been synthesized as mean and standard deviation or median, minimum and maximum. The Fisher test for qualitative variables and the Kruskal-Wallis test or the one-way ANOVA technique for quantitative variables was calculated. The hypotheses of normality and homoschedasticity have been verified through the Shapiro-Wilk test and the Bartlett test. All the tests were considered significant for relative values p < 0.05. Furthermore, odds ratios (OR) with 95% confidence intervals (CI) and their significance were calculated.

## Supplementary information

Supplementary Figure 1
