# Supplementary material for: Regulation of PDE5 expression in human aorta and thoracic aortic aneurysms
Source: Sci Rep. 2019 Aug 21;9:12206. doi: 10.1038/s41598-019-48432-6 (PMC6704119; doi:10.1038/s41598-019-48432-6)

Regulation of PDE5 expression in human aorta and thoracic aortic aneurysms

Valeriana Cesarini
Calogera Pisano
Gabriele Rossi
Carmela Rita Balistreri
Flavia Botti
Giorgio Antonelli
Giovanni Ruvolo
Emmanuele A. Jannini
Susanna Dolci

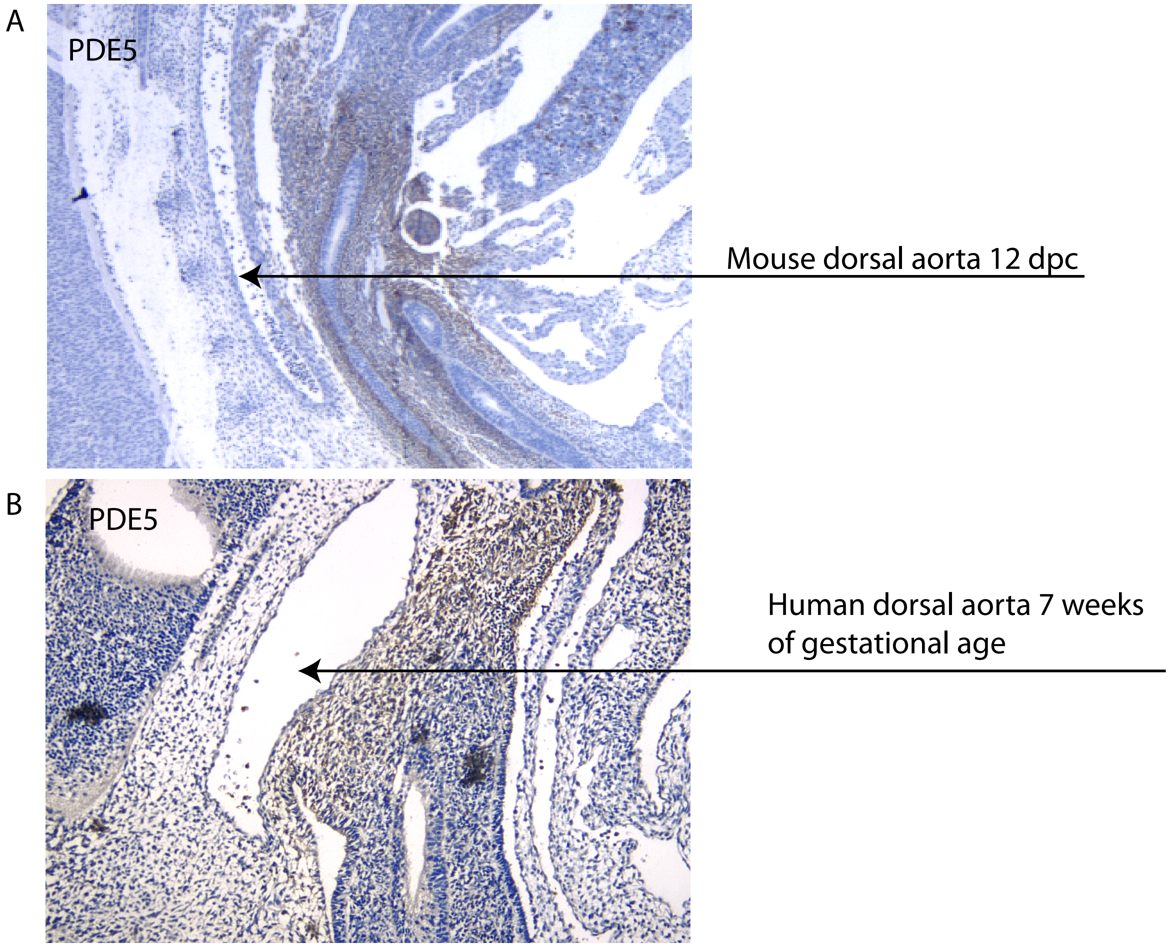

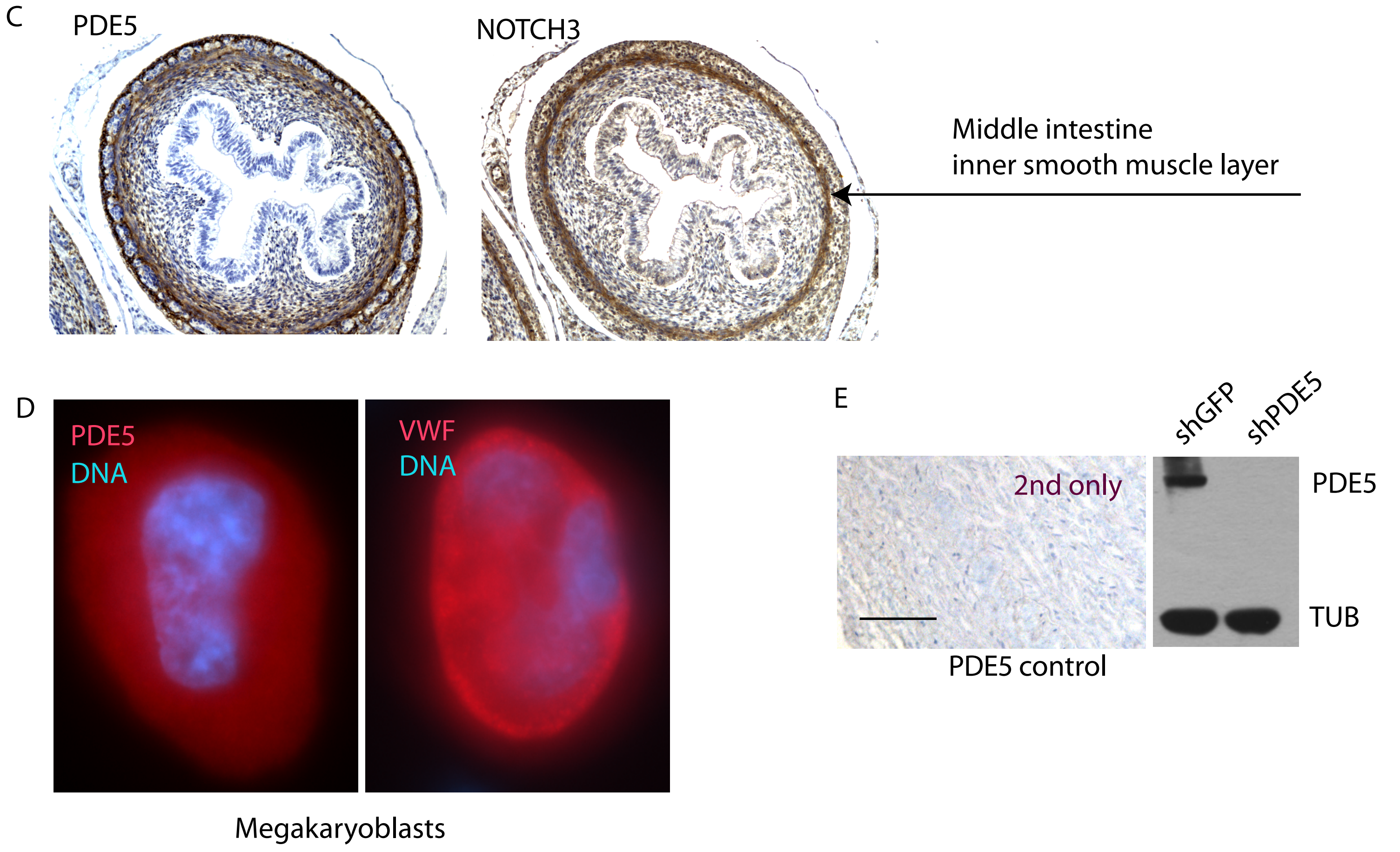

Supplement: Supplementary file 1 — Supplementary Figure 1 [file 41598_2019_48432_MOESM1_ESM.pdf]
